# Large cortical bone pores in the tibia are associated with proximal femur strength

**DOI:** 10.1371/journal.pone.0215405

**Published:** 2019-04-17

**Authors:** Gianluca Iori, Johannes Schneider, Andreas Reisinger, Frans Heyer, Laura Peralta, Caroline Wyers, Melanie Gräsel, Reinhard Barkmann, Claus C. Glüer, J. P. van den Bergh, Dieter Pahr, Kay Raum

**Affiliations:** 1 Berlin-Brandenburg Center for Regenerative Therapies, Charité –Universitätsmedizin Berlin, corporate member of Freie Universität Berlin, Humboldt-Universität zu Berlin, and Berlin Institute of Health, Berlin, Germany; 2 Division Biomechanics, Karl Landsteiner University of Health Sciences, Krems, Austria; 3 Department of Internal Medicine, NUTRIM School of Nutrition and Translational Research in Metabolism, Maastricht University Medical Center, Maastricht, The Netherlands; 4 Department of Internal Medicine, VieCuri Medical Center, Venlo, The Netherlands; 5 Laboratoire d’Imagerie Biomédicale, Sorbonne Universités, INSERM UMR S 1146, CNRS UMR 7371, Paris, France; 6 Department of Biomedical Engineering, School of Biomedical Engineering & Imaging Sciences, King’s College London, London, United Kingdom; 7 Sektion Biomedizinische Bildgebung, Klinik für Radiologie und Neuroradiologie, Christian-Albrechts-Universität zu Kiel, Kiel, Germany; 8 Institute for Lightweight Design and Structural Biomechanics, TU Wien, Vienna, Austria; University of Notre Dame, UNITED STATES

## Abstract

Alterations of structure and density of cortical bone are associated with fragility fractures and can be assessed in vivo in humans at the tibia. Bone remodeling deficits in aging women have been recently linked to an increase in size of cortical pores. In this ex vivo study, we characterized the cortical microarchitecture of 19 tibiae from human donors (aged 69 to 94 years) to address, whether this can reflect impairments of the mechanical competence of the proximal femur, i.e., a major fracture site in osteoporosis. Scanning acoustic microscopy (12 μm pixel size) provided reference microstructural measurements at the left tibia, while the bone vBMD at this site was obtained using microcomputed tomography (microCT). The areal bone mineral density of both left and right femoral necks (aBMD_neck_) was measured by dual‐energy X‐ray absorptiometry (DXA), while homogenized nonlinear finite element models based on high-resolution peripheral quantitative computed tomography provided hip stiffness and strength for one-legged standing and sideways falling loads. Hip strength was associated with aBMD_neck_ (r = 0.74 to 0.78), with tibial cortical thickness (r = 0.81) and with measurements of the tibial cross-sectional geometry (r = 0.48 to 0.73) of the same leg. Tibial vBMD was associated with hip strength only for standing loads (r = 0.59 to 0.65). Cortical porosity (Ct.Po) of the tibia was not associated with any of the femoral parameters. However, the proportion of Ct.Po attributable to large pores (diameter > 100 μm) was associated with hip strength in both standing (r = -0.61) and falling (r = 0.48) conditions. When added to aBMD_neck_, the prevalence of large pores could explain up to 17% of the femur ultimate force. In conclusion, microstructural characteristics of the tibia reflect hip strength as well as femoral DXA, but it remains to be tested whether such properties can be measured in vivo.

## Introduction

With >3.5 million fragility fractures annually in Europe only, osteoporosis represents a significant burden on the society [1]. In elderly subjects, the hip is the most frequent and severe osteoporotic fracture site [2]. In a population of increasing age, hip fractures represent a dramatic cause of functional decline, morbidity and mortality [3,4]. Despite these facts, a large number of hip fractures occurs in patients without diagnosed osteoporosis [5]. The failure in detecting alterations of the cortical bone microstructure is considered one of the reasons of the only modest efficacy of the current DXA-based fracture risk assessment [6–8]. In an attempt to fill this diagnostic gap, studies have investigated the association of structural features in cortical bone with fracture risk [9,10]. One motivation for this has been the observation that in long bones, a reduction of the cortical thickness (Ct.Th) and an increase in the cortical porosity (Ct.Po) are responsible for the larger part of the age-related bone loss [11]. Fueled by the advent of new technology such as high-resolution peripheral quantitative computed tomography (HR-pQCT), which allows the imaging of the distal skeleton in vivo with a spatial resolution down to 95 μm, clinical studies have associated Ct.Po and Ct.Th of the tibia and radius of humans with age, disease, fracture history, treatment and training [12–18].

Recent work on morphological alterations of bone multicellular units (BMUs) have extended our understanding of the way in which the microstructure of cortical bone is affected by aging. The age-related uncoupling between bone resorption and formation has been associated with prolonged osteoclastic activity and delayed refilling of resorption cavities in cortical bone [19]. As a consequence, cortical bone pores progressively increase in size and tend to coalesce, as recently observed in iliac crest specimens [20]. Interestingly, similar (large, irregular) cavities have been observed in femoral neck biopsies obtained from patients undergoing joint replacement following hip fracture [21].

Since osteoporosis occurs systemically throughout the skeleton, pore morphological changes are likely to be reflected in peripheral bones, which can be assessed in vivo more easily than the proximal femur. In a clinical study on Type 2 diabetes patients, a larger cortical pore diameter (Po.Dm) and increased diameter heterogeneity were observed at the distal skeleton of fractured subjects when compared to controls [15]. The increased Po.Dm at the distal site of both tibia and radius was accompanied by a significant increase of Ct.Po and by a reduction of the predicted strength of these bones, even though statistical significance was reached only for the distal sites of patients with Diabetes Mellitus. Backed by these findings, we hypothesized that enlarged cortical pores in the peripheral skeleton might reflect an impairment of the mechanical competence of the hip, a site of major relevance for fracture.

Ex vivo studies have investigated the association between the cortical bone of the tibia and the fracture load of human femur samples as early as 1996 [22], but rarely considered features of cortical pore morphology. One recent work has combined mechanical testing with HR-pQCT of tibia samples [23]. The authors reported strong correlations between properties of the distal tibia (total vBMD and simulated strength) and the strength of vertebrae and of proximal femora from the same donor. The microstructure of cortical bone, however, was not considered. Studies that took cortical microarchitecture into account have only included cortical porosity (Ct.Po) as single structural parameter [24].

The aim of this work was to quantify the correlation between the architecture of tibial cortical bone (macro- and microscopic, with an emphasis on variations of pore morphology), with the stiffness and strength of proximal femur samples. The analysis of the cortical bone microstructure was performed on the anteromedial tibia shaft, since this region represents a favorable site for in vivo ultrasound measurements [25]. We also asked whether cortical bone properties at the tibia are able to explain the mechanical competence of the hip alternatively or in addition to DXA.

## Materials and methods

### Samples

The lower limbs of nineteen human donors were collected at the Anatomy Institute of the Lübeck University. The scientific use of human tissue from body donors is permitted by the German law “Gesetz über das Leichen-, Bestattungsund Friedhofswesen des Landes Schleswig-Holstein—Abschnitt II, §9 (Leichenöffnung, anatomisch)” from 04.02.2005. The donors have agreed to scientific use of their bodies.

Left and right femora were stored, while only the left tibiae were available for the lower leg. All bone specimens were dissected and frozen at -20°C until and between experiments. The average donor age was 84 ± 8 years (69–94 years; 6 male, 13 female). Incomplete or no information was available regarding the medical history of the subjects. Proximal femur samples were prepared by cutting and embedding the diaphysis 80 mm below the lesser trochanter, as described elsewhere [26]. During dissection, the distal portion of the tibia samples had been already removed. The exact proportion of shaft missing was estimated to vary between 25% and 60%.

### DXA

DXA measurements of all (left and right) proximal femur samples were performed after dissection and removal of the soft tissues on a Hologic Discovery A scanner (Discovery QDR, Hologic Inc., USA). During the scan, the samples were immersed in 14 cm-deep saline solution in order to simulate soft tissue attenuation. The areal BMD of the femoral neck (aBMD_neck_) was measured from the projection of the bone on the coronal plane.

### HR-pQCT

The 38 proximal femora were thawed, fixed in a custom-made plastic chamber [27], submerged in 1% PBS, degassed, and scanned using an XtremeCT II scanner (Scanco Medical AG, Brüttisellen, Switzerland). X-ray tube voltage and current were set to 68 kVp and 1470 μA, respectively. Images were acquired using an integration time of 200 ms and by taking 3000 projections over 180°. The reconstruction led to stacks of 4608 × 4608 images with an isotropic voxel size of 30.3 μm. For the conversion of voxel integers to bone mineral density (BMD), the scanner built-in calibration rule was used.

### Finite element based mechanical testing

**Fig 1:**
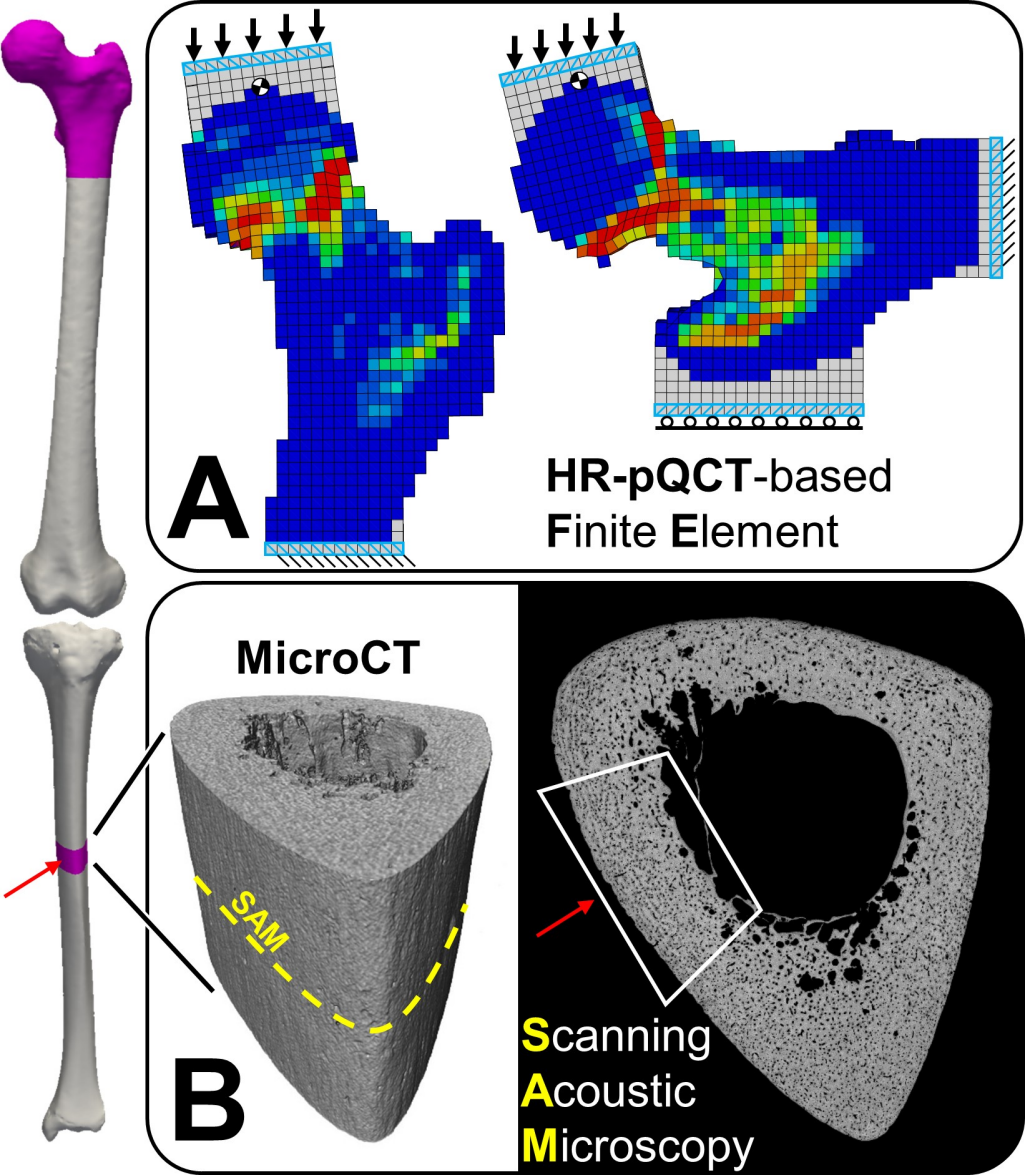
Summary of materials and methods. (A) HR-pQCT-based finite element models were developed to compute (left and right) hip stiffness and strength under loading conditions representative of one-legged stance and of a sideways fall. (B) MicroCT and SAM images from a cross-section of the left tibia midshaft (19.5 ± 3.8 cm away from the knee) of the same donors are used to characterize density and architecture of cortical bone. Microstructural measurements are obtained from a region of the bone that can be reached in vivo by diagnostic ultrasound (red arrow).

Non-linear homogenized voxel FE models of the proximal femur were developed from the 38 HR-pQCT datasets following an already described procedure [26]. Briefly, the HR-pQCT volume was first coarsened with a factor 10, yielding an isotropic voxel size of 0.303 mm. Voxels of residual air bubbles were set to the gray value of water as obtained from the intensity histogram of the entire scan. Volumes were further coarsened to an isotropic voxel size of 2.7 mm (Fig 1A), and gray values converted first to vBMD and then to bone volume fraction. For this, a linear calibration rule was derived for the specific set of samples using 3D registered scanning acoustic microscopy (SAM) and HR-pQCT images of the proximal femur shafts [28]. An elastic-yield constitutive law based on the local bone volume fraction was adapted, as described in [26]. This implements a piecewise Hill criterion with different yield stresses for compression and tension [29]. Asymmetric material (elastic and yield) constants for the model were taken from an experimental study on trabecular bone samples and extrapolated for cortical bone by use of a monotonic scaling function as described elsewhere [26,30]. The failure of each bone was simulated during one-legged standing (STANCE: 20° inclination in the frontal plane; Panel A in S1 Fig) as well as during a sideways fall (FALL: 0° internal rotation, 30° adduction angle; Panel B in S1 Fig).The models were prepared using medtool 4.1 (Dr. Pahr Ingenieurs e.U, Pfaffstätten, Austria) and solved in Abaqus 6.12 (Simulia, Dassault Systemes, Velizy, France). Stiffness (hvFE_S) and strength (hvFE_Fu) of each proximal femur were calculated for both loading conditions. The proximal femora from 10 out of 19 donors were selected for biomechanical failure tests (S1 Fig). Experiments were performed according to an established protocol [26], and provided validation for the results (hvFE_S and hvFE_Fu) of the FE simulations (S1 Section and S1 Fig).

### Micro CT

The midshaft portion of each left tibia was cut and positioned in the field of view of a small animal microCT system (VivaCT 80; Scanco Medical, Brüttisellen, Switzerland). A custom thermo-isolated plastic cylinder filled with dry ice was used to keep the sample frozen while scanning and the shaft axis was aligned with the rotation axis of the cylinder holder. X-ray tube voltage and current were set to 70 kV and 114 μA, respectively. 500 projections were taken over 360° of rotation and with an exposure time of 200 ms. The field of view had a length of 70 mm and was reconstructed as a stack of 1024 × 1024 voxels images with an isotropic voxel size of 39 μm. The volume data was filtered with a Gaussian smoothing kernel (σ = 1.1 voxels, radius = 2.0 voxels) and Hounsfield units were converted to vBMD based on the calibration procedure provided by the manufacturer.

### Scanning acoustic microscopy

**Fig 2:**
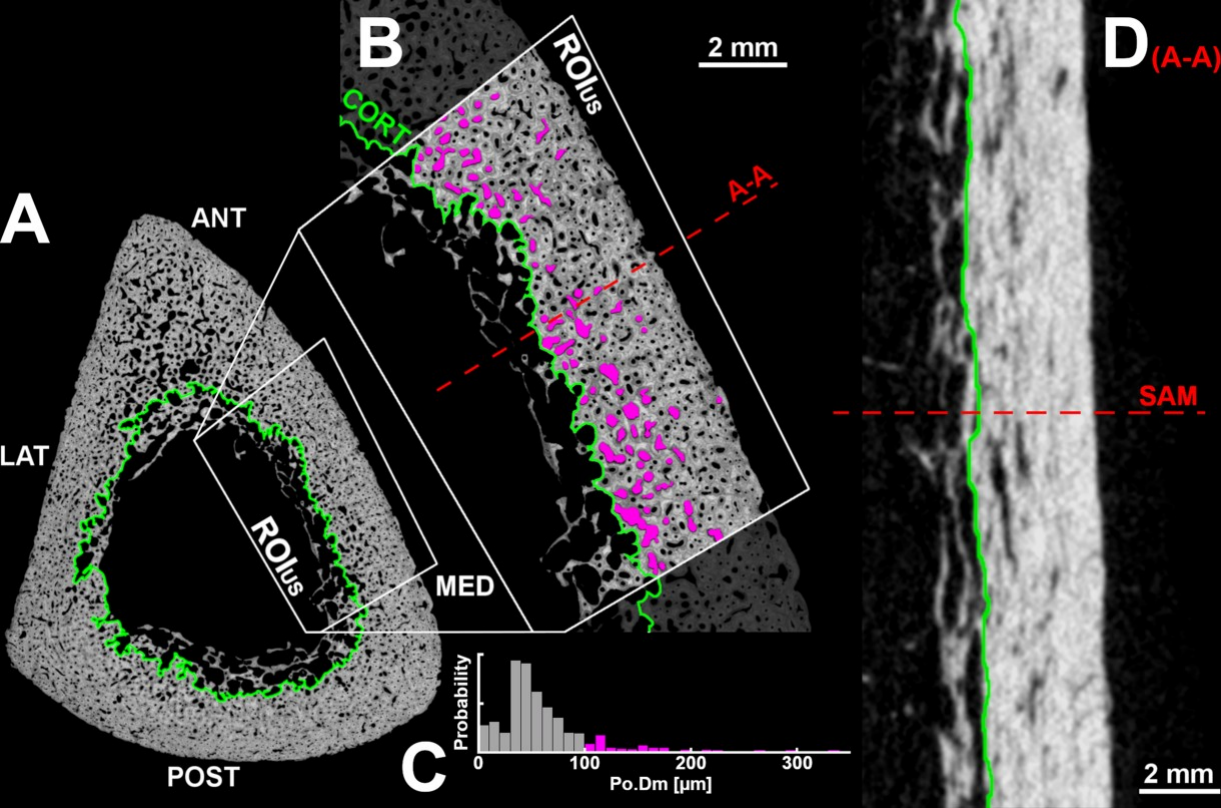
SAM and microCT image processing. (A) SAM cross section with endosteal boundary marked in green. (B) Anteromedial detail of A, with ROI_*US*_ highlighted: this region can be reached in vivo by ultrasound waves. A total number of 11.932 cortical bone pores were analyzed from the ROI_*US*_ of all samples. Cortical bone pores with diameter (Po.Dm) > 100 *μ*m are colored in magenta. (C) Pore size distribution within the ROI_*US*_ of B: the tail (Po.Dm > 100 *μ*m) of the histogram represents 53% of the total cortical bone porosity. (D) 20-mm longitudinal microCT section centered through the ROI_*US*_.

Transversal cross sections (21 mm in height) were extracted using a precision band saw (EXACT GmbH, Remscheid, Germany) from the region of the tibia shaft imaged with microCT and at a distance of 19.5 ± 3.8 cm from the proximal end of the bone. After washing, the proximal surface of each cross section was ground and polished on a planar grinder (Phoenix 4000, Buehler Ltd., Illinois) at a constant speed of 50 rpm and with decreasing grain size (ISO/FEPA grit: P80, P600, P1200, P2500 and P4000, Buehler Ltd., Illinois). After polishing, the samples were washed again, submerged in 1% PBS and degassed inside a desiccator for at least 30 min to remove air bubbles from the cortical pores. The scans were performed in 1% degassed PBS solution at a controlled temperature of 25°C, using a custom acoustic microscope described elsewhere [31,32]. The transducer (KSI 100/60°, KSI, Herborn, Germany) had a central frequency of 100 MHz, a -6 dB depth of focus of 139 μm and a diameter of the focused ultrasound beam of 19.8 μm in the focal plane [32]. Signals were processed to obtain calibrated acoustic impedance maps with a pixel size of 12 μm [32] (Fig 1B and Fig 2).

### Image processing

#### MicroCT

A 20 mm-thick portion of the microCT volume centered on the SAM image plane was processed. Voxels belonging to the bone tissue were segmented using Otsu’s method [33]. 3D masks of the cortical bone compartment were computed with the algorithm described by Burghardt et al. [34] For this, the threshold radius for filling of large pores had to be increased to 2.0 mm for the two samples with highest porosity. A binary image of the whole tibia bone was obtained by tracing the external boundary of the cortical bone mask automatically.

#### SAM

An adaptive threshold was applied to separate the bone tissue from the background of the SAM images [35]. Afterwards, the bone tissue mask was cleaned by first removing unconnected objects with area below 0.144 mm^2^, and subsequent filling of all single-pixel pores. The endosteal boundary was drawn manually, following a set of rules described elsewhere [36] (Fig 2A and 2B). The periosteal contour was automatically traced on a morphologically closed version of the bone tissue mask (radius of the structuring element = 0.48 mm). Cortical bone porosity (Ct.Po) and the diameter of single Haversian Canals (Po.Dm) were measured on a binary mask of the pores. To investigate the relative contribution of large to giant [37,38] cortical pores on the total pore number and on cortical bone porosity, Po.Dm thresholds of 60, 100, 160, 300 and 385 μm were used (Fig 2C). The cortical bone microstructure was characterized on SAM images from the anteromedial region of the shaft (ROI_US_; Figs 1 and 2), since this area represents the target of in vivo measurements with ultrasound. The SAM image processing pipeline is available online at: https://doi.org/10.5281/zenodo.2605365 and can be reproduced by downloading the original SAM images from: https://doi.org/10.5281/zenodo.2605350.

Table 1 presents abbreviations and a description of all parameters measured from microCT and SAM images.

**Table 1:** Bone properties of the tibia midshaft measured with microCT and SAM.

	Name	Unit	Description
**microCT**			
***vBMD***_***tot***_	Bone mineral density	[mgHA/cm^3^]	Of the entire bone
***vBMD***_***cort***_			Of the cortical bone
**SAM**			
***Tt*.*Ar***	Total area	[mm^2^]	Area occupied by the bone cross section
***Ct*.*Ar***	Cortical area	[mm^2^]	Area of cortical bone
***T*.*Ar***	Tissue area	[mm^2^]	Area of the bone tissue
***Ct*.*Wba***	Areal portion of cortical tissue	[%]	Cortical tissue area / Tt.Ar
***Ct*.*Th***	Cortical thickness	[mm]	Most frequent minimum distance between peri- and endosteal surfaces
***Ct*.*Po***	Cortical porosity	[%]	100 × (1 –tissue pixels / cortical bone pixels)
***Po*.*D***	Pore density	[#/mm^2^]	Number of pores per square mm
***relPo*.*n***_***60***_μ_***m***_	Prevalence of large pores	[%]	Number of pores with diameter larger than a fixed threshold divided by total number of pores
***Po*.*Dm***	Pore diameter	[mm]	Diameter of the largest inscribed circle [20]
***Po*.*Dm***_***10%***_	Po.Dm quantiles	[mm]	Quantiles of the Po.Dm distribution
***relCt*.*Po***_***60***_μ_***m***_	Relative proportion of porosity	[%]	Proportion of porosity due to pores with diameter above fixed threshold

### Statistical analysis

Distributions of single variables were tested for normality using Shapiro-Wilk tests. A paired t-test was used to compare left and right aBMD_neck_ from DXA as well as hvFE_S and hvFE_Fu. Associations between aBMD_neck_ or tibial cortical bone and hvFE_S and hvFE_Fu were investigated by linear regression analysis (Pearson’s r). Linear regressions were investigated between left tibia properties and separately (i) left and (ii) averaged left and right femoral hvFE_S and hvFE_Fu. Linear partial correlation was used to measure the association between tibial cortical bone and hip stiffness and strength after controlling for aBMD_neck_. The adjusted R^2^ of multivariate linear models of hvFE_S and hvFE_Fu was characterized when adding one microstructural covariate to aBMD_neck_. All image and statistical analyses were performed in Matlab (R2018a, The Mathworks Inc., Natick, MA, USA). Results were considered statistically significant for p < 0.05.

## Results

### Proximal femur densitometry and mechanics

Proximal femur aBMD_*neck*_ and mechanical properties (hvFE_S and hvFE_Fu) are summarized in Table *2*. The distribution of the differences between left and right aBMD_*neck*_ values had a mean that did not significantly differ from zero. The same was the case between left and right hvFE_S and hvFE_Fu in STANCE. For FALL simulations, left and right femora showed modest but significant differences in hvFE_S and hvFE_Fu. The results of STANCE FE simulations were in very good agreement with values from biomechanical tests (R^2^ = 0.95, p < 0.0001 and R^2^ = 0.89, p < 0.0001 for hvFE_S and hvFE_Fu, respectively; Panels C and D in S1 Fig). FALL simulations showed good agreement with experimental strength (R^2^ = 0.86, p < 0.0001; Panel F in S1 Fig) and moderate agreement with experimental stiffness (R^2^ = 0.68, p = 0.003; Panel E in S1 Fig).

**Table 2:** Results from DXA and FE simulations.

	Whole sample (n = 38)	left (n = 19)	right (n = 19)
**DXA**			
**aBMD**_**neck**_ **[mgHA/cm^2^]**	532 ± 102 (380–760)	529 ± 96 (404–760)	534 ± 110 (380–755)
**FE simulations**			
** STANCE**			
** hvFE_S [N/mm]**	3394 ± 1400 (1310–6889)	3210 ± 1343 (1310–6664)	3578 ± 1468 (1536–6889)
** hvFE_Fu [N]**	2582 ± 927 (1243–4926)	2605 ± 903 (1367–4926)	2558 ± 974 (1243–4860)
** FALL**			
** hvFE_S [N/mm]**	1221 ± 370 (616–2071)	1314 ± 376 (817–2071)	1127 ± 348 (616–1946)
** hvFE_Fu [N]**	1372 ± 449 (655–2691)	1456 ± 460 (851–2691)	1289 ± 434 (655–2405)

hvFE_S, homogenized voxel finite element proximal femur stiffness; hvFE_Fu, homogenized voxel finite element proximal femur ultimate force; STANCE, physiological one-legged standing; FALL, sideways fall.

### Structure and density of the tibia midshaft

Volumetric BMD and structural properties of the cortical bone of the tibia are summarized in Table 3, together with inter-sample coefficients of variation and correlations with aBMD_neck_, hvFE_S and hvFE_Fu from the same leg. 95% Confidence Intervals (CIs) of the Pearson r’s of Table 3 are collected in S3 Table.

**Table 3:** Hip DXA, macroscopic geometry and vBMD of the tibia midshaft, architecture and composition of tibial cortical bone.

								control for aBMD_neck_			
				STANCE		FALL		STANCE		FALL	
			aBMD_neck_	hvFE_S	hvFE_Fu	hvFE_S	hvFE_Fu	hvFE_S	hvFE_Fu	hvFE_S	hvFE_Fu
	Mean ± SD (min-max)	CV [%]	Pearson r								
**Left hip (n = 19)**											
**DXA**											
**aBMD**_**neck**_ **[mgHA/cm^2^]**	529 ± 96 (404–760)	18	/	0.62*	0.74**	0,66*	0,78**	/	/	/	/
**Left tibia (n = 19)**											
**MicroCT (whole cross section)**											
**vBMD**_**tot**_ **[mgHA/cm^3^]**	617 ± 133 (261–776)	22	0.46	0.69*	0.65*			0.58	0.52		
**vBMD**_**cort**_ **[mgHA/cm^3^]**	914 ± 54 (801–988)	6		0.72**	0.63*			0.65*	0.53		
**SD(vBMD**_**cort**_**) [mgHA/cm^3^]**	185 ± 36 (131–266)	19		-0.66*	-0.59*			-0.62*	-0.54		
**SAM (whole cross section)**											
**Tt.Ar [mm^2^]**	441 ± 110 (326–829)	26									
**Ct.Ar [mm^2^]**	238 ± 65 (77–349)	25	0.51	0.59*	0.71**	0,58	0,60*		0.58		
**T.Ar [mm^2^]**	235 ± 59 (96–333)	22	0.47	0.52	0.67*	0,57	0,60*		0.55		
**Ct.Wba [%]**	49.1 ± 14.5 (15.6–69.8)	27	0.51	0.76**	0.73**		0,48	0.65*	0.61*		
**SAM (ROI**_**US**_**)**											
**Ct.Th [mm]**	2.98 ± 1.19 (0.82–5.35)	40	0.75**	0.66*	0.81**	0,77**	0,81**		0.57	0.56	0.54
**Ct.Po [%]**	11.1 ± 3.6 (7.7–21.4)	32									
**Po.D [1/mm^2^]**	16.9 ± 1.8 (13.2–21.1)	11									
**Po.D**_**60μm**_ **[1/mm^2^]**	4.5 ± 1.1 (2.8–6.2)	25									
**Po.D**_**100μm**_ **[1/mm^2^]**	1.3 ± 0.7 (0.5–3.4)	56		-0.54	-0.56				-0.52		
**Po.D**_**160μm**_ **[1/mm^2^]**	0.3 ± 0.3 (0.1–1.4)	94		-0.52	-0.52			-0.49	-0.54		
**relPo.n**_**60**_μ_**m**_ **[%]**	27.9 ± 6.7 (18.0–38.4)	24									
**relPo.n**_**100**_μ_**m**_ **[%]**	7.6 ± 4.3 (2.5–20.9)	56		-0.53	-0.57			-0.47	-0.56		
**relPo.n**_**160**_μ_**m**_ **[%]**	1.9 ± 1.8 (0.4–8.5)	96		-0.51	-0.52			-0.49	-0.56		
**Po.Dm [mm]**	51 ± 6 (44–67)	12			-0.47			ns	ns		
**SD(Po.Dm) [mm]**	34 ± 7 (23–55)	21		-0.55	-0.57			-0.52	-0.60*		
**Po.Dm**_**10%**_ **[mm]**	19 ± 4 (12–25)	20									
**Po.Dm**_**90%**_ **[mm]**	91 ± 19 (68–152)	21		-0.49	-0.54				-0.51		
**Ct.Po**_**60**_μ_**m**_ **[%]**	7.9 ± 3.6 (4.5–18.9)	46		-0.46	-0.50				-0.48		
**Ct.Po**_**100**_μ_**m**_ **[%]**	4.8 ± 3.5 (1.5–16.4)	73		-0.50	-0.52				-0.51		
**Ct.Po**_**160**_μ_**m**_ **[%]**	2.4 ± 2.6 (0.4–11.4)	107			-0.47				-0.50		
**relCt.Po**_**60**_μ_**m**_ **[%]**	68.9 ± 8.6 (54.8–88.3)	13		-0.51	-0.60*	-0,49	-0,50		-0.60*		
**relCt.Po**_**100**_μ_**m**_ **[%]**	40.1 ± 13.9 (17.3–77.0)	35		-0.61*	-0.63*	-0,46	-0,48	-0.54	-0.62*		
**relCt.Po**_**160**_μ_**m**_ **[%]**	18.9 ± 12.1 (5.1–53.6)	64		-0.50	-0.53				-0.54		

The last nine columns show the Pearson coefficients of the linear correlation with aBMDneck, hvFE_S and hvFE_Fu and the Pearson r of the linear partial correlation analysis controlling for the effect of aBMD_neck_, for both STANCE and FALL loading conditions. Coefficients are reported only for p-values < 0.05. The 95% Confidence Intervals for the correlation coefficients of this table can be found in S3 Table.

* p < 0.01

** p < 0.001.

**Fig 3:**
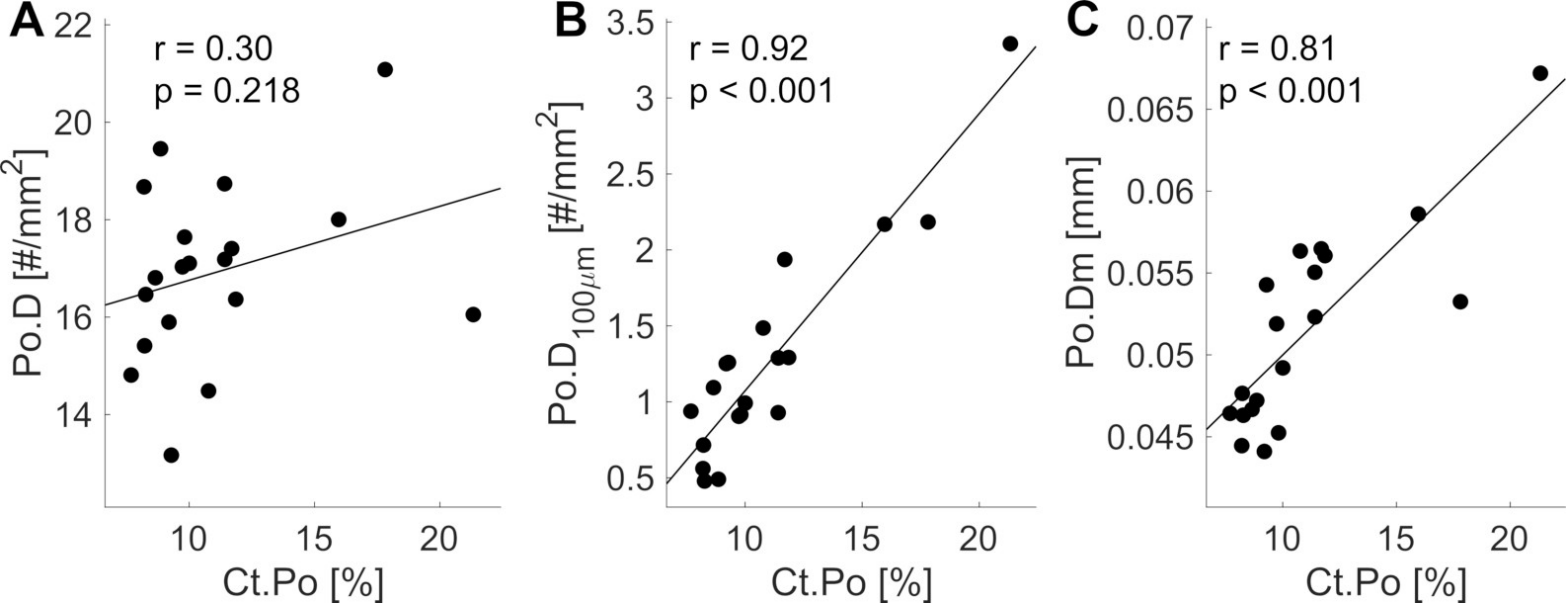
Cortical bone microstructure of the anteromedial tibia in association with Ct.Po. Ct.Po is independent from the density of canals (A). Its increase is largely explained by an increase of the density of large pores (B) or of the mean pore diameter (C).

Between the 19 investigated tibiae, cortical bone exhibited large variability in Ct.Th (CV = 40%) and Ct.Po (CV = 32%), modest variations in pore density (Po.D; CV = 11%), and almost invariant vBMD values (CV = 6%). Remarkably, pore density was not associated with Ct.Po (Fig 3A). On the contrary, the density of pores with a diameter larger than 100 μm showed higher inter-sample variability (CV = 56%) and was strongly correlated with Ct.Po (r = 0.92, p < 0.001; Fig 3B). Ct.Po was also correlated with the average Po.Dm (r = 0.81, p < 0.001; Fig 3C). Large pores (diameter > 100 μm) were mainly observed at the endosteal side (Fig 2B) and despite representing only the 7.6% of all the evaluated pores, they contributed, on average, to 40% of the total sample Ct.Po.

### Correlation of tibial cortical bone and aBMD_neck_ with femoral stiffness and strength of the same leg

**Fig 4:**
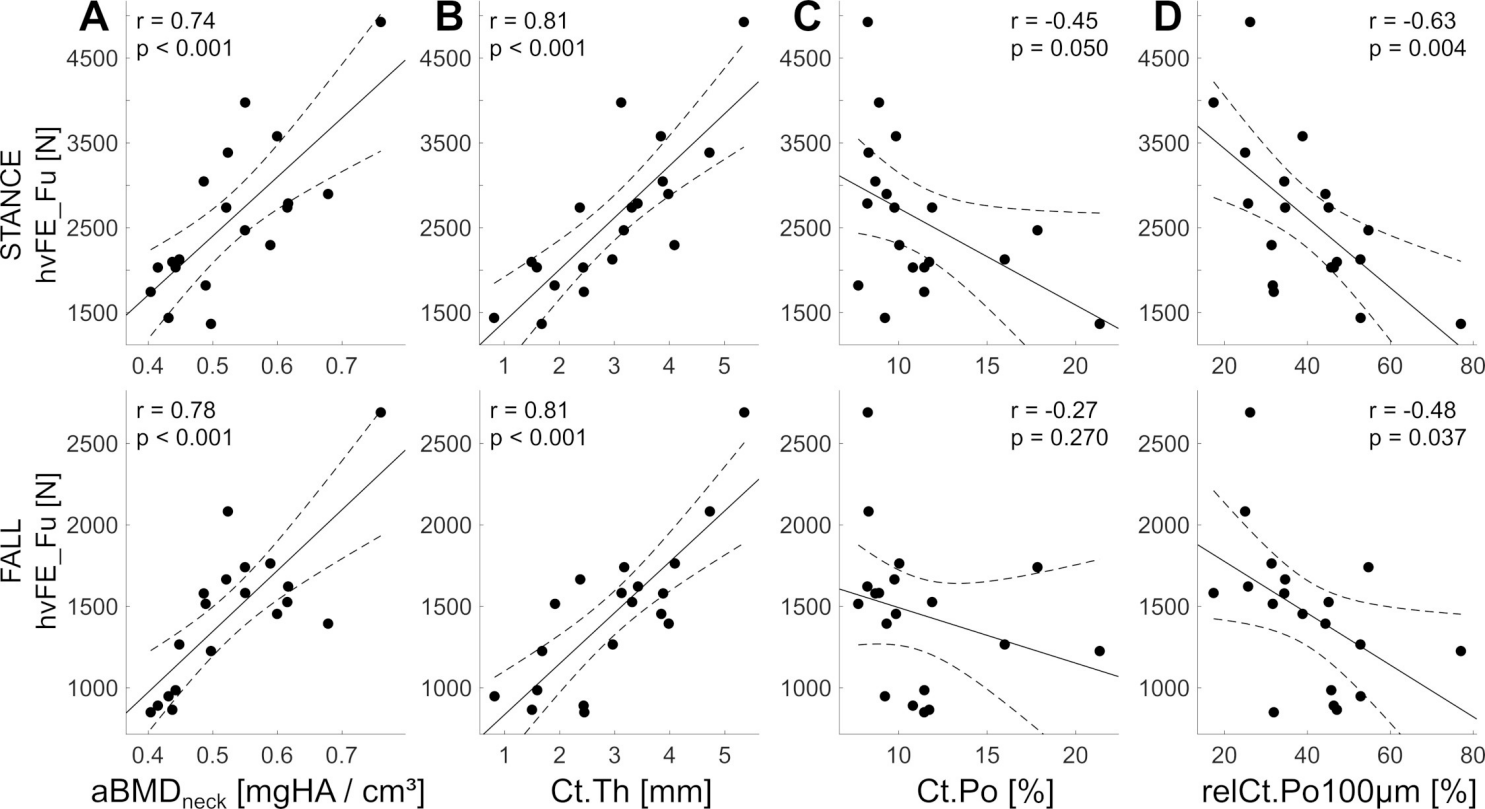
Associations with proximal femur mechanical competence. Linear regression between DXA aBMD at the femur neck (A) as well as whole tibia cortical thickness (B), intracortical porosity (C) and relative porosity due to large pores (diameter > 100 μm) in the anteromedial tibia (D) with the FE-based femoral strength under standing and sideways falling loads.

As expected, aBMD_neck_ was associated with both proximal femur hvFE_S (r = 0.62 and 0.66 for STANCE and FALL, respectively; both p < 0.01) and hvFE_Fu (r = 0.74 and 0.78 for STANCE and FALL, respectively; p < 0.001, Fig 4A).

Descriptors of the tibial cross-sectional geometry and total vBMD were only moderately correlated to aBMD_neck_ (0.46 ≤ r ≤ 0.51, p < 0.05; Table 3). The correlation between aBMD_neck_ and Ct.Th was strong (r = 0.75, p < 0.001). No association was found between aBMD_neck_ and the pore microstructure in the tibia.

The mineral density of the tibia was associated with hip stiffness (r = 0.69, p < 0.01 and r = 0.72, p < 0.001 for vBMD_tot_ and vBMD_cort_, respectively) and strength (r = 0.65 and r = 0.63 for vBMD_tot_ and vBMD_cort_, respectively, both p < 0.01).

Cortical bone area (Ct.Ar), bone tissue area (T.Ar) and areal portion of cortical bone (Ct.Wba) of the tibia were associated with variations of the hip hvFE_S and hvFE_Fu when measured both in stance and fall conditions (Table 3).

The cortical thickness of the tibia showed strong associations with the stiffness (r = 0.66, p < 0.01 for STANCE and r = 0.77, p < 0.001 for FALL) and strength (r = 0.81, p < 0.001 for both STANCE and FALL; Fig 4B). Ct.Po did not show significant correlations with the mechanical properties of the hip (Fig 4C).

There was a clear negative association between parameters describing the density and prevalence of large pores (diameter > 100 μm) with variations of hvFE_S and hvFE_Fu in STANCE (Table 3). The relative contribution of large pores to Ct.Po (relCt.Po_100_μ_m_) was associated with the hip mechanics in both STANCE (r = -0.61 and r = -0.63 for hvFE_S and hvFE_Fu, respectively; both p < 0.01) and FALL (r = -0.46 and r = -0.48 for hvFE_S and hvFE_Fu, respectively; both p < 0.05) loads (regressions with hvFE_Fu are plotted in Fig 4D). Except for relCt.Po, parameters of the pore microstructure did not show significant associations for FALL loads. Po.Dm thresholds are reported only until 160 μm since larger thresholds did not provide significant associations (data not shown).

### Multivariate models of proximal femur stiffness and strength

After controlling for aBMD_neck_, the degree of association between tibia measurements and the mechanical properties of the proximal femur was generally reduced (last four columns of Table 3). Parameters of the pore morphology maintained a similar degree of association with hvFE_Fu in STANCE even after controlling for the effect of aBMD_neck_.

Linear combinations of aBMD_neck_ and relCt.Po_100_μ_m_ had adjusted R^2^ values that were 17% and 16% larger than those of models of aBMD_neck_ alone, for hvFE_S and hvFE_Fu, respectively, but this pattern was limited to standing loads (Table 4). The combination of aBMD_neck_ and Ct.Th did not improve the correlation with hvFE_S and hvFE_Fu, if compared to Ct.Th alone.

**Table 4:** Multivariate regression models of proximal femur stiffness and strength.

n = 19		STANCE						FALL					
		hvFE_S			hvFE_Fu			hvFE_S			hvFE_Fu		
		beta	p-val	R^2^	beta	p-val	R^2^	beta	p-val	R^2^	beta	p-val	R^2^
y = a × **Ct.Th** + b		0.88	2e-3	0.40	0.73	3e-5	0.63	0.29	1e-4	0.57	0.37	3e-5	0.63
y = a × **aBMD**_**neck**_ + b		0.83	5e-3	0.34	0.67	3e-4	0.52	0.25	2e-3	0.41	0.36	9e-5	0.58
y = a × **aBMD**_**neck**_ + …		0.63	0.01	0.51	0.54	6e-4	0.68						
	… b × **relCt.Po**_**100**_μ_**m**_ + c	-0.61	0.02		-0.40	6e-3							

Standardized coefficients (beta), p-values and adjusted R^2^ are reported only for multivariate models that showed a significant increase of stiffness or ultimate force prediction if compared to single parameter ones.

## Discussion

In this work, we asked whether the cortical bone of the tibia can reflect changes in the stiffness and fracture resistance of the hip.

### vBMD, thickness and presence of large pores in tibial cortical bone are associated with hip stiffness and strength

We found significant associations between the vBMD and structure of the tibia midshaft with the stiffness and ultimate force of the proximal femur as predicted by non-linear, homogenized finite element analysis. The cortical thickness of the tibia showed strong associations with the proximal femur strength, with correlation coefficients comparable to those obtained with a DXA scan.

The heterogeneity and the tail of the Po.Dm distribution were negatively associated with proximal femur stiffness and strength when these were measured by STANCE simulations, pointing out the important role of large cortical pores under physiological loading conditions. Interestingly, the associations between tibial Ct.Po and proximal femur mechanics were significant only when Ct.Po was calculated from the 7.6% of pores with larger diameter, and the same trend was observed for pore density. In a recent report, Ct.Po from the same (anteromedial) region of the tibia diaphysis measured here was associated (r = -0.50) with the proximal femur strength by mechanical tests in standing conditions [24]. Even if the correlation was not significant in our study (p = 0.05 for tibial Ct.Po and hvFE_S, with n = 19, whereas n = 28 in Abraham et al.), both works report high variability for the hip strength of legs with low tibial Ct.Po (see the left half (Ct.Po < 15%) of the plots of Fig 4C). Our data suggest that cases with impaired hip strength could be further distinguished by analyzing the contribution of abnormally large pores on the total Ct.Po of the tibia. This finding is not in contrast with in vivo reports on the association between fracture risk and Ct.Po as measured by HR-pQCT, since the imaging of cortical pores with HR-pQCT is in a way “tuned” towards the detection of large cavities due to the resolution limit of the scanner (i.e. 130 μm and 95 μm for 1^st^ and 2^nd^ generation HR-pQCT, respectively). HR-pQCT can estimate Ct.Po beyond its nominal resolution by using BMD-based approaches [28], meaning that a measurement of relCt.Po is readily available in vivo from HR-pQCT images. Therefore, future HR-pQCT studies should investigate the relation between fracture risk and the prevalence of large pores in the cortical bone of the distal skeleton.

The occurrence of large pores weakens the mechanical resistance of cortical bone. Osteonal diameter has been shown to be negatively associated with cortical bone toughness [39,40], whereas large endosteal pores can increase the strain energy density in the surrounding bone tissue during a compression of the fibula [41]. Local clustering of large and progressively opening cavities have been suggested as a possible causes of regional instability of the femur neck [21,37]. Besides this, the prevalence of pores with abnormal size is a fingerprint of age-induced alterations of bone remodeling, in which Haversian canals drift towards coalescing and partially non-refilled resorption units [19,20]. Our results suggest that the observation of such morphological changes of cortical pores in the tibia of living humans might reveal an impairment of the proximal femur mechanical competence.

### Pore size reflects proximal femur strength independently of DXA

Macroscopic changes of vBMD, cortical bone area and thickness at the tibia midshaft had associations with proximal femur mechanics that could in large part be accounted for by a measurement of aBMD_neck_. On the contrary, changes of the pore microstructure were independent of aBMD_neck_, and adding this information substantially improved the prediction of femur strength obtained by DXA. This suggests that hip strength information provided by measurements of the tibial geometry and vBMD is largely redundant, if acquired in addition to a DXA scan. In contrast, measurements of the pore microarchitecture at the tibia might convey hip strength information which is not captured by aBMD. It should be noted, however, that our results allow this conclusion exclusively for hip strength during one-legged standing, a configuration representing only minor fracture risk [42].

### The anteromedial tibia is a favorable site for assessment of the pore microstructure

In a recent report, the hip failure load has been reported to be associated with low vBMD and microstructural alterations of the distal tibia, as assessed (ex vivo) using an HR-pQCT protocol for in vivo scans [23]. In comparison, our results showed significant associations between tibial vBMD and the ultimate force of the proximal femur only for physiological standing loads. Possible reasons for this discrepancy are the different scan regions and the different spatial resolutions. Kroker et al. measured the total vBMD at the distal portion of the tibia, supposedly capturing information from both trabecular and cortical bone density. On the contrary, the midshaft region scanned in our study contains predominantly cortical bone. For comparison, vBMD_tot_ ranges were 261–776 mgHA/cm^3^ and 52–332 mgHA/cm^3^ in our and Kroker’s study, respectively, confirming the different type of bone tissue considered for the two vBMD measurements. Towards the epiphyses of the tibia, cortical bone becomes thinner and is increasingly replaced by a trabecular core, rising concerns about the precision error of cortical bone structural and density measurements performed at distal and ultradistal sites with HR-pQCT [43,44]. Due to the different measurement site (midshaft, here, instead of distal shaft) we observed a cortical thickness of the tibia that was 2 to 3 times larger than values reported from HR-pQCT studies [15,18,45–47]. In this sense, the tibia midshaft provided a much larger and homogeneous volume of interest for cortical bone microstructural characterization than the distal shaft. Ultrasound waves represent an ionizing radiation free alternative for cortical bone characterization and can non-invasively be transmitted to and along bone at the facies medialis of the tibia midshaft, where the periosteum is covered by a thin layer of soft tissue. At this location, novel quantitative ultrasound techniques can measure thickness, speed of sound and porosity of cortical bone in vivo [25,48,49]. Our findings indicate the relevance of microstructural measurements performed at the facies medialis of the tibia for the prediction of the proximal femur strength. To confirm the advantage of this specific ROI, we repeated all microstructural measurements considering the entire tibia cross section (S1 Table). The degree of association with the hip stiffness and strength was not changed and the same microstructural features (Ct.Th and prevalence of large pores) remained relevant.

Finally, we addressed the clinical scenario in which a subject’s hip strength is predicted based on a measurement performed on a single leg by performing regression analyses between properties of the left tibia and the average hvFE_S and hvFE_Fu of left and right femora (S2 Table). This confirmed the relevance of all parameters identified by the left tibia–left hip regressions (i.e. tibia geometry and Ct.Th for both STANCE and FALL loads; tibia vBMD and large pores for STANCE loads). The geometry and Ct.Th of the tibia, however, were less affected by the anatomical side of the correlation, whereas the Pearson r of correlations between pore microstructure and hvFE_S and hvFE_Fu was reduced, on average, by 11.3% and 11.9%, respectively.

### Study limitations

The current study presents several limitations. The characterization of the cortical bone microarchitecture was performed on 2D SAM images with a resolution of about 20 μm. Despite this, Ct.Po and Po.Dm values were in very good agreement with 3D gold-standard synchrotron-CT measurements conducted at the diaphysis of the tibia and femur [38,46,50]. Compared to SAM, microCT overestimated Ct.Ar (p < 0.01), T.Ar (p < 0.001), Ct.Wba (p < 0.001) and Ct.Th (p = 0.04) (S4 Table). The 3D Ct.Th obtained with microCT from a 20 mm-thick shaft section was 6.7% to 11.5% larger than Ct.Th assessed from single 2D cross-sectional SAM images, likely due to the different ways in which the separation between trabecular and cortical bone compartments is obtained for SAM and microCT. Despite this, macroscopic structural properties and Ct.Th obtained from microCT and SAM were in very good agreement (R^2^ = 0.89 to 0.99; S4 Table).

Considering the ROI for density and microstructural assessments in the tibia, it was not possible to standardize its location along the axis of the diaphysis: the tibiae were measured at a distance from their proximal end that varied between 12.2 cm to 27.2 cm, representing a possible source of error. This was necessary because a portion (between 25% and 60%) of the tibia had been removed during dissection. Despite this kind of variability, our data showed significant relationships with the mechanics of the proximal femur, suggesting that measurements of the tibia remain valuable even under such conditions. In vivo, protocols for the consistent positioning of the measurement ROI should be followed, as is done in pQCT and HR-pQCT procedures [43].

This work used quasi-static homogenized voxel FE models to simulate the mechanical stiffness and strength of 38 human proximal femora. We dedicated a subset of 20 samples to biomechanical testing and replicated the FE validation published in 2013 by Dall’Ara et al., obtaining FE accuracy for strength and for standing stiffness comparable to values from the literature [26,51,52], whereas the lower accuracy for stiffness in FALL could be explained by the poor contact between bone and embedding during the initial loading phase (S1 Section). The displacement rate applied in our experiment was constant and several orders of magnitude smaller than what is expected at the proximal femur or measured at the pelvis during a sideways fall [53,54]. In a recent comparison between fall and fixed displacement rate experiments, Gilchrist et al. reported significant differences between the ultimate force for the two test modalities [55]. Their findings, however, had low statistical power, were dependent on the displacement rate itself and were relevant only for the ultimate force, but not for the proximal femur stiffness. For our purposes, the choice of quasi-static loading was taken in the light of the comparison between biomechanical tests and an already validated FE procedure [26]. Homogenized non-linear quasi-static FE simulations provide accurate predictions of the proximal femur ultimate force, stiffness, fracture energy and location obtained by quasi-static as well as dynamic sideways fall experiments [26,56], supporting the validity of our findings also for higher strain rates.

It should be noted, finally, that microstructural measurements at the tibia of human donors were performed by means of SAM and microCT: two modalities that cannot be used for the examination of tibia properties in living subjects. However, the tibia midshaft can be imaged in vivo both, by 2^nd^ generation HR-pQCT and by US. The ability of these techniques to provide microstructural predictors of hip strength will require further confirmation.

## Conclusion

Recent evidence on intracortical bone remodeling have shown that an age-induced delay in osteoprogenitor recruitment following pore resorption leads to a progressive enlargement and accumulation of cavities in cortical bone [20]. In this ex vivo study, the contribution to cortical porosity of canals with a diameter larger than 100 μm in the tibia of human donors was associated with reduced strength and stiffness of the proximal femur. The cortical bone of the tibia represents a key diagnostic opportunity for the prediction of the bone fracture risk since it is load bearing and can be measured in vivo by HR-pQCT and ultrasound. Our results indicate that cortical bone thickness and the prevalence of large voids in tibial cortical bone should be taken into account as biomarkers of a mechanical impairment of the hip, alternatively or in addition to standard DXA metrics.

## Supporting information

S1 Section FE model validation. (DOC)

S1 Fig Mechanical test setup and FE model validation. (A) Mechanical test setup for STANCE, showing a detail of the 20° inclination in the frontal plane. (B) FALL mechanical tests (0° internal rotation, 30° adduction angle). The load direction is contained in the plane defined by the femoral neck and shaft axes. (C) Association between finite element predictions and biomechanical measurements of proximal femur stiffness (R^2^ = 0.95, p < 0.0001) and (D) strength (R^2^ = 0.89, p < 0.0001) for STANCE. (E) Association between finite element predictions and biomechanical measurements of proximal femur stiffness (R^2^ = 0.68, p < 0.001) and (D) strength (R^2^ = 0.86, p < 0.0001) for FALL.
(TIF)

S1 Table Whole tibia microstructure. Microstructure of the whole cross-section of the tibia midshaft from SAM, together with Pearson coefficients of the linear correlation with aBMDneck, hvFE_S and hvFE_Fu.
(DOC)

S2 Table Comparison with the subject’s hip stiffness and strength. Correlation coefficients between LEFT tibia properties and hvFE_S and hvFE_Fu calculated as the average between LEFT and RIGHT femora, together with the relative change with respect to the LEFT-LEFT regression.
(DOC)

S3 Table Confidence intervals for Table 2. Confidence intervals of the coefficients of correlation between tibial cortical bone vBMD and architecture with aBMD_neck_ and proximal femur stiffness and strength.
(DOC)

S4 Table Geometry and cortical thickness of the tibia midshaft from microCT. Mean, Standard Deviation, ranges and coefficient of variation (CV) are reported for each variable together with the R^2^ and the p-value of the comparison (paired t-test or Wilcoxon signed rank test when parameters were not normally distributed) with the corresponding SAM measurement. For Cross-sectional areal properties for microCT are calculated dividing the corresponding volumetric measurement by the height (20 mm) of the analyzed stack.
(DOC)
