# Correction: Large cortical bone pores in the tibia are associated with proximal femur strength

**DOI:** 10.1371/journal.pone.0219443

**Published:** 2019-07-05

**Authors:** Gianluca Iori, Johannes Schneider, Andreas Reisinger, Frans Heyer, Laura Peralta, Caroline Wyers, Melanie Gräsel, Reinhard Barkmann, Claus C. Glüer, J. P. van den Bergh, Dieter Pahr, Kay Raum

There are errors in Tables 1 and 3. Please see the correct Tables 1 and 3 here.

**Table 1:** Bone properties of the tibia midshaft measured with microCT and SAM.

	Name	Unit	Description
**microCT**			
***vBMD***_***tot***_	Bone mineral density	[mgHA/cm³]	Of the entire bone
***vBMD***_***cort***_			Of the cortical bone
**SAM**			
***Tt*.*Ar***	Total area	[mm²]	Area occupied by the bone cross section
***Ct*.*Ar***	Cortical area	[mm²]	Area of cortical bone
***T*.*Ar***	Tissue area	[mm²]	Area of the bone tissue
***Ct*.*Wba***	Areal portion of cortical tissue	[%]	Cortical tissue area / Tt.Ar
***Ct*.*Th***	Cortical thickness	[mm]	Most frequent minimum distance between peri- and endosteal surfaces
***Ct*.*Po***	Cortical porosity	[%]	100 × (1 –tissue pixels / cortical bone pixels)
***Po*.*D***	Pore density	[#/mm²]	Number of pores per square mm
***relPo*.*n***_***60µm***_	Prevalence of large pores	[%]	Number of pores with diameter larger than a fixed threshold divided by total number of pores
***Po*.*Dm***	Pore diameter	[µm]	Diameter of the largest inscribed circle [20]
***Po*.*Dm***_***10%***_	Po.Dm quantiles	[µm]	Quantiles of the Po.Dm distribution
***relCt*.*Po***_***60µm***_	Relative proportion of porosity	[%]	Proportion of porosity due to pores with diameter above fixed threshold

**Table 3:** Hip DXA, macroscopic geometry and vBMD of the tibia midshaft, architecture and composition of tibial cortical bone.

								control for aBMD_neck_			
				STANCE		FALL		STANCE		FALL	
			aBMD_neck_	hvFE_S	hvFE_Fu	hvFE_S	hvFE_Fu	hvFE_S	hvFE_Fu	hvFE_S	hvFE_Fu
	**Mean ± SD (min-max)**	**CV [%]**	**Pearson r**								
**Left hip (n = 19)**											
**DXA**											
**aBMD**_**neck**_ **[mgHA/cm²]**	529 ± 96 (404–760)	18	/	0.62^*^	0.74^**^	0,66^*^	0,78^**^	/	/	/	/
**Left tibia (n = 19)**											
**MicroCT (whole cross section)**											
**vBMD**_**tot**_ **[mgHA/cm³]**	617 ± 133 (261–776)	22	0.46	0.69^*^	0.65^*^			0.58	0.52		
**vBMD**_**cort**_ **[mgHA/cm³]**	914 ± 54 (801–988)	6		0.72^**^	0.63^*^			0.65^*^	0.53		
**SD(vBMD**_**cort**_**) [mgHA/cm³]**	185 ± 36 (131–266)	19		-0.66^*^	-0.59^*^			-0.62^*^	-0.54		
**SAM (whole cross section)**											
**Tt.Ar [mm²]**	441 ± 110 (326–829)	26									
**Ct.Ar [mm²]**	238 ± 65 (77–349)	25	0.51	0.59^*^	0.71^**^	0,58	0,60^*^		0.58		
**T.Ar [mm²]**	235 ± 59 (96–333)	22	0.47	0.52	0.67^*^	0,57	0,60^*^		0.55		
**Ct.Wba [%]**	49.1 ± 14.5 (15.6–69.8)	27	0.51	0.76^**^	0.73^**^		0,48	0.65^*^	0.61^*^		
**SAM (ROI**_**US**_**)**											
**Ct.Th [mm]**	2.98 ± 1.19 (0.82–5.35)	40	0.75^**^	0.66^*^	0.81^**^	0,77^**^	0,81^**^		0.57	0.56	0.54
**Ct.Po [%]**	11.1 ± 3.6 (7.7–21.4)	32									
**Po.D [1/mm²]**	16.9 ± 1.8 (13.2–21.1)	11									
**Po.D**_**60µm**_ **[1/mm²]**	4.5 ± 1.1 (2.8–6.2)	25									
**Po.D**_**100µm**_ **[1/mm²]**	1.3 ± 0.7 (0.5–3.4)	56		-0.54	-0.56				-0.52		
**Po.D**_**160µm**_ **[1/mm²]**	0.3 ± 0.3 (0.1–1.4)	94		-0.52	-0.52			-0.49	-0.54		
**relPo.n**_**60µm**_ **[%]**	27.9 ± 6.7 (18.0–38.4)	24									
**relPo.n**_**100µm**_ **[%]**	7.6 ± 4.3 (2.5–20.9)	56		-0.53	-0.57			-0.47	-0.56		
**relPo.n**_**160µm**_ **[%]**	1.9 ± 1.8 (0.4–8.5)	96		-0.51	-0.52			-0.49	-0.56		
**Po.Dm [µm]**	51 ± 6 (44–67)	12			-0.47			ns	ns		
**SD(Po.Dm) [µm]**	34 ± 7 (23–55)	21		-0.55	-0.57			-0.52	-0.60^*^		
**Po.Dm**_**10%**_ **[µm]**	19 ± 4 (12–25)	20									
**Po.Dm**_**90%**_ **[µm]**	91 ± 19 (68–152)	21		-0.49	-0.54				-0.51		
**Ct.Po**_**60µm**_ **[%]**	7.9 ± 3.6 (4.5–18.9)	46		-0.46	-0.50				-0.48		
**Ct.Po**_**100µm**_ **[%]**	4.8 ± 3.5 (1.5–16.4)	73		-0.50	-0.52				-0.51		
**Ct.Po**_**160µm**_ **[%]**	2.4 ± 2.6 (0.4–11.4)	107			-0.47				-0.50		
**relCt.Po**_**60µm**_ **[%]**	68.9 ± 8.6 (54.8–88.3)	13		-0.51	-0.60^*^	-0,49	-0,50		-0.60^*^		
**relCt.Po**_**100µm**_ **[%]**	40.1 ± 13.9 (17.3–77.0)	35		-0.61^*^	-0.63^*^	-0,46	-0,48	-0.54	-0.62^*^		
**relCt.Po**_**160µm**_ **[%]**	18.9 ± 12.1 (5.1–53.6)	64		-0.50	-0.53				-0.54		

The last nine columns show the Pearson coefficients of the linear correlation with aBMDneck, hvFE_S and hvFE_Fu and the Pearson r of the linear partial correlation analysis controlling for the effect of aBMD_neck_, for both STANCE and FALL loading conditions. Coefficients are reported only for p-values < 0.05. The 95% Confidence Intervals for the correlation coefficients of this table can be found in S3 Table.

* p < 0.01;

** p < 0.001.
