# Supplementary material for: Large cortical bone pores in the tibia are associated with proximal femur strength
Source: PLoS One. 2019 Apr 17;14(4):e0215405. doi: 10.1371/journal.pone.0215405 (PMC6469812; doi:10.1371/journal.pone.0215405)
Supplement: S1 Section — (DOC) [file pone.0215405.s001.doc]

To validate the results from the FE simulations, biomechanical tests were performed, on left and right proximal femora from 10 out of 19 donors, following the protocol established by Dall’Ara et al. in 2013 [1]. One side (randomly selected) was mounted in one-legged standing (STANCE) configuration with 20° inclination in the frontal plane (Panel B in S1 Fig). The counter proximal femur was prepared for mechanical testing simulating a sideways fall (FALL: 0° internal rotation, 30° adduction angle; Panel B in S1 Fig). The load was applied on the femoral head and in the plane defined by the femoral neck and shaft axes at a rate of 5 mm/min until failure and while recording a video of the anterior side of the sample (PowerShot SX160 IS, Canon, Japan). The axial force was measured using a 100 kN load cell (U3 force transducer, HBM, Germany). The 3D displacements of the setup and of the bone sample were measured using active infrared markers (Optotrak Certus, Northern Digital Inc., Canada). At the moment of the test, each sample had been subjected to 3 freezing-thawing cycles. The experimental femoral strength (Exp_Fu) was defined as the ultimate force recorded during the experiment, while the femoral stiffness (Exp_S) was defined as the maximum slope recorded during at least 20 % of the load–displacement curve [2]. For this, the displacements measured by the infrared markers on the setup were used [1].

For STANCE, the agreement between FE-based and experimentally measured mechanical properties was excellent for both stiffness (R² = 0.95, SEE = 270 N/mm, Panel C in S1 Fig) and ultimate force (R² = 0.89, SEE = 800 N, Panel D in S1 Fig).

For sideways FALL testing, FEM and mechanical testing agreed very well on ultimate load (R² = 0.86, SEE = 309 N, Panel F in S1 Fig), but the correlation was only moderate for stiffness (R² = 0.68, SEE = 197 N/mm, Panel E in S1 Fig). The videos of the experiment and the recordings of the infrared markers suggested a poor contact between bone and PU embedding at the greater trochanter during the initial loading phase. The vertical displacement of the sample inside the mold during the first stage of the test might explain the discrepancy between simulated and experimentally measured stiffness in FALL.

1. Dall’Ara E, Luisier B, Schmidt R, Kainberger F, Zysset P, Pahr D. A nonlinear QCT-based finite element model validation study for the human femur tested in two configurations in vitro. Bone. 2013;52: 27–38. doi:10.1016/j.bone.2012.09.006

2. Benca E, Reisinger A, Patsch JM, Hirtler L, Synek A, Stenicka S, et al. Effect of simulated metastatic lesions on the biomechanical behavior of the proximal femur. J Orthop Res. 2017;35: 2407–2414. doi:10.1002/jor.23550
