# Supplementary figures and images for: Large cortical bone pores in the tibia are associated with proximal femur strength
Source: PLoS One. 2019 Apr 17;14(4):e0215405. doi: 10.1371/journal.pone.0215405 (PMC6469812; doi:10.1371/journal.pone.0215405)

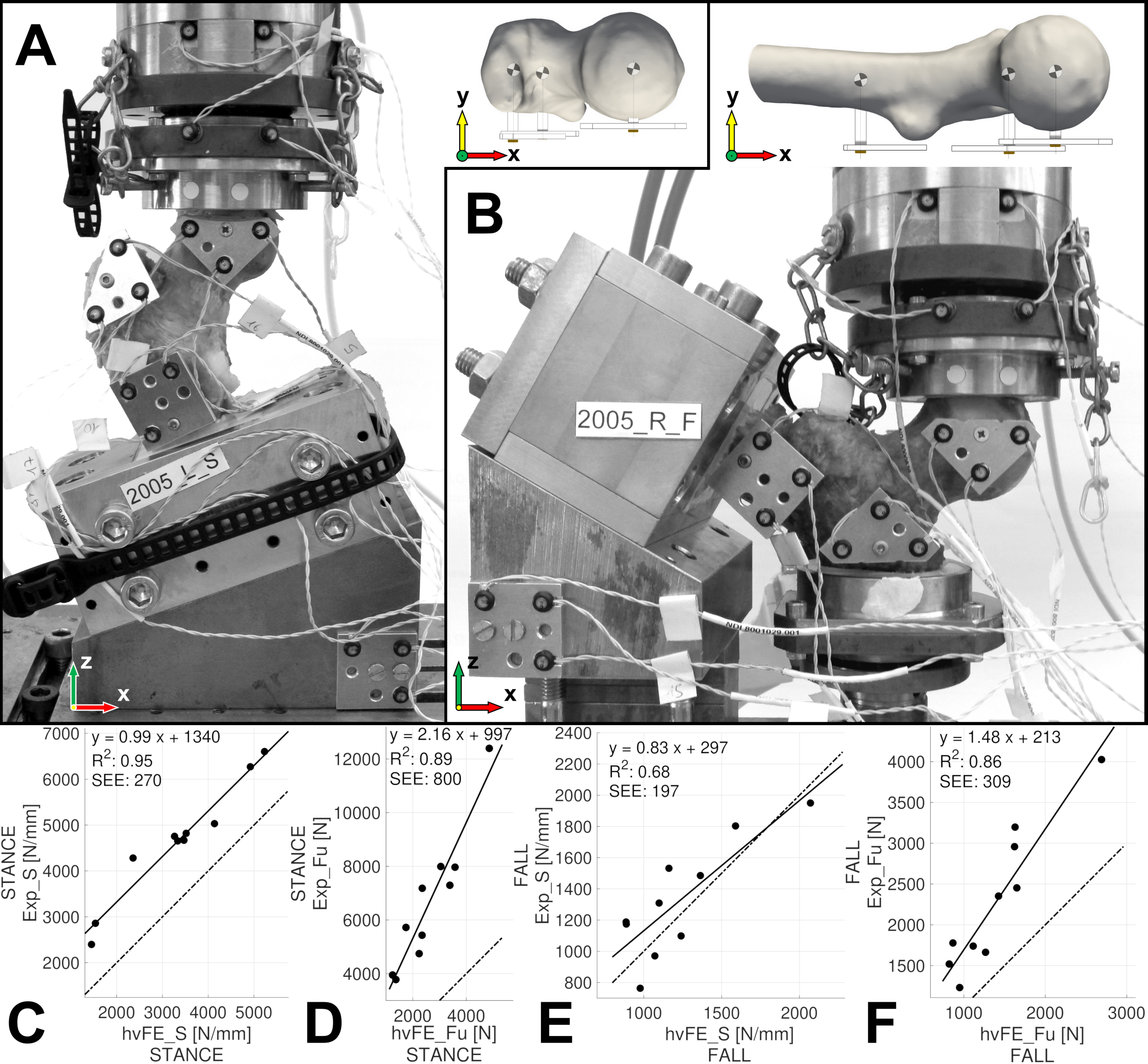

Supplement: S1 Fig — (A) Mechanical test setup for STANCE, showing a detail of the 20° inclination in the frontal plane. (B) FALL mechanical tests (0° internal rotation, 30° adduction angle). The load direction is contained in the plane defined by the femoral neck and shaft axes. (C) Association between finite element predictions and biomechanical measurements of proximal femur stiffness (R2 = 0.95, p < 0.0001) and (D) strength (R2 = 0.89, p < 0.0001) for STANCE. (E) Association between finite element predictions and biomechanical measurements of proximal femur stiffness (R2 = 0.68, p < 0.001) and (D) strength (R2 = 0.86, p < 0.0001) for FALL. (TIF) [file pone.0215405.s002.tif]
