# Supplementary material for: Large cortical bone pores in the tibia are associated with proximal femur strength
Source: PLoS One. 2019 Apr 17;14(4):e0215405. doi: 10.1371/journal.pone.0215405 (PMC6469812; doi:10.1371/journal.pone.0215405)
Supplement: S1 Table — Microstructure of the whole cross-section of the tibia midshaft from SAM, together with Pearson coefficients of the linear correlation with aBMDneck, hvFE_S and hvFE_Fu. (DOC) [file pone.0215405.s003.doc]

|  | | | | STANCE | | FALL | |
| --- | --- | --- | --- | --- | --- | --- | --- |
|  | | | aBMD_neck_ | hvFE_S | hvFE_Fu | hvFE_S | hvFE_Fu |
| SAM (whole cross section) (n=19) | Mean ± SD (min-max) | CV [%] | Pearson r | | | | |
| Ct.Th [mm] | 3.11 ± 1.27 (0.79-5.55) | 41 | 0.61^*^ | 0.79^**^ | 0.85^**^ | 0.50 | 0.65^*^ |
| Ct.Po [%] | 11.7 ± 4.0 (7.4-24.9) | 9 |  |  |  |  |  |
| Po.D [1/mm²] | 16.1 ± 1.4 (13.8-19.3) | 9 |  |  |  |  |  |
| Po.D_60µm_ [1/mm²] | 4.4 ± 1.1 (2.4-6.3) | 24 |  |  |  |  |  |
| Po.D_100µm_ [1/mm²] | 1.3 ± 0.7 (0.3-3.3) | 51 |  | -0.52 | -0.50 |  |  |
| Po.D_160µm_ [1/mm²] | 0.4 ± 0.3 (0.0-1.5) | 84 |  | -0.49 | -0.47 |  |  |
| relPo.n_60µm_ [%] | 28.8 ± 7.4 (15.4-41.5) | 26 |  |  |  |  |  |
| relPo.n_100µm_ [%] | 8.4 ± 4.8 (1.6-24.0) | 57 |  | -0.51 | -0.49 |  |  |
| relPo.n_160µm_ [%] | 2.4 ± 2.2 (0.2-10.5) | 93 |  | -0.47 |  |  |  |
| Po.Dm [mm] | 53 ± 7 (43-74) | 14 |  |  | -0.46 |  |  |
| SD(Po.Dm) [mm] | 36 ± 9 (21-64) | 24 |  | -0.51 | -0.47 |  |  |
| Po.Dm_10%_ [mm] | 19 ± 3 (12-25) | 16 |  |  |  |  |  |
| Po.Dm_90%_ [mm] | 94 ± 20 (68-162) | 22 |  | -0.47 | -0.46 |  |  |
| Ct.Po_60µm_ [%] | 8.7 ± 4.3 (4.0-22.9) | 49 |  | -0.48 | -0.47 |  |  |
| Ct.Po_100µm_ [%] | 5.6 ± 4.3 (1.0-20.6) | 76 |  | -0.47 | -0.46 |  |  |
| Ct.Po_160µm_ [%] | 3.1 ± 3.4 (0.2-15.6) | 107 |  |  |  |  |  |
| relCt.Po_60µm_ [%] | 71.5 ± 9.7 (49.5-92.0) | 14 |  | -0.58^*^ | -0.54 |  |  |
| relCt.Po_100µm_ [%] | 43.7 ± 15.2 (12.5-82.9) | 35 |  | -0.57 | -0.52 |  |  |
| relCt.Po_160µm_ [%] | 23.0 ± 13.5 (2.7-62.8) | 59 |  | -0.51 | -0.50 |  |  |

Coefficients are reported only for p-values < 0.05. * p < 0.01; ** p < 0.001.
