# Supplementary material for: Large cortical bone pores in the tibia are associated with proximal femur strength
Source: PLoS One. 2019 Apr 17;14(4):e0215405. doi: 10.1371/journal.pone.0215405 (PMC6469812; doi:10.1371/journal.pone.0215405)
Supplement: S2 Table — Correlation coefficients between LEFT tibia properties and hvFE_S and hvFE_Fu calculated as the average between LEFT and RIGHT femora, together with the relative change with respect to the LEFT-LEFT regression. (DOC) [file pone.0215405.s004.doc]

|  |  | | **STANCE** | | | | **FALL** | | | |
| --- | --- | --- | --- | --- | --- | --- | --- | --- | --- | --- |
|  | **aBMD_neck_** | | **hvFE_S** | | **hvFE_Fu** | | **hvFE_S** | | **hvFE_Fu** | |
|  | **Pearson r (% change with respect to LEFT-LEFT regression)** | | | | | | | | | |
| **Left hip DXA (n=19)** | | | | | | | | | | |
| **aBMD_neck_ [mgHA/cm²]** | / | | 0.63^*^ | (+1.7%) | 0.72^**^ | (-2.0%) | 0.66^*^ | (+0.0%) | 0.77^**^ | (-0.7%) |
| **Left tibia (n=19)** | | | | | | | | | | |
| **vBMD_tot_ [mgHA/cm³]** | 0.49 | (+6.4%) | 0.68^*^ | (-1.7%) | 0.61^*^ | (-5.9%) |  |  |  |  |
| **vBMD_cort_ [mgHA/cm³]** |  |  | 0.67 | (-7.3%) | 0.57 | (-8.8%) |  |  |  |  |
| **SD(vBMD_cort_) [mgHA/cm³]** |  |  | -0.57 | (-13.6%) | -0.52 | (-12.4%) |  |  |  |  |
| **Tt.Ar [mm²]** |  |  |  |  |  |  |  |  |  |  |
| **Ct.Ar [mm²]** | 0.51 | (+1.9%) | 0.56 | (-5.2%) | 0.68^*^ | (-3.7%) | 0.66^*^ | (+14.4%) | 0.67^*^ | (+11.9%) |
| **T.Ar [mm²]** | 0.49 | (+5.1%) | 0.50 | (-5.1%) | 0.65^*^ | (-3.5%) | 0.65^*^ | (+15.2%) | 0.67^*^ | (+12.3%) |
| **Ct.Wba [%]** | 0.53 | (+2.5%) | 0.74^**^ | (-2.6%) | 0.69^*^ | (-6.2%) |  |  | 0.52 | (+9.2%) |
| **ROI_US_** | | | | | | | | | | |
| **Ct.Th [mm]** | 0.74^**^ | (-0.8%) | 0.65^*^ | (-1.2%) | 0.76^**^ | (-5.4%) | 0.76^**^ | (-1.5%) | 0.79^**^ | (-2.5%) |
| **Ct.Po [%]** |  |  |  |  |  |  |  |  |  |  |
| **Po.D [1/mm²]** |  |  |  |  |  |  |  |  |  |  |
| **Po.D_60µm_ [1/mm²]** |  |  |  |  |  |  |  |  |  |  |
| **Po.D_100µm_ [1/mm²]** |  |  | -0.46 | (-14.8%) | -0.48 | (-14.2%) |  |  |  |  |
| **Po.D_160µm_ [1/mm²]** |  |  |  | ns | ns | |  |  |  |  |
| **relPo.n_60µm_ [%]** |  |  |  |  |  |  |  |  |  |  |
| **relPo.n_100µm_ [%]** |  |  | -0.46 | (-12.2%) | -0.50 | (-11.9%) |  |  |  |  |
| **relPo.n_160µm_ [%]** |  |  |  | ns | -0.46 | (-12.7%) |  |  |  |  |
| **Po.Dm [mm]** |  |  |  |  | ns | |  |  |  |  |
| **SD(Po.Dm) [mm]** |  |  | -0.47 | (-13.6%) | -0.49 | (-14.3%) |  |  |  |  |
| **Po.Dm_10%_ [mm]** |  |  |  |  |  |  |  |  |  |  |
| **Po.Dm_90%_ [mm]** |  |  | ns | | -0.47 | (-13.1%) |  |  |  |  |
| **Ct.Po_60µm_ [%]** |  |  | ns | | ns | |  |  |  |  |
| **Ct.Po_100µm_ [%]** |  |  | ns | | ns | |  |  |  |  |
| **Ct.Po_160µm_ [%]** |  |  |  |  | ns | |  |  |  |  |
| **relCt.Po_60µm_ [%]** |  |  | ns | | -0.52 | (-13.1%) | -0.50 | (+1.9%) | -0.49 | (-1.5%) |
| **relCt.Po_100µm_ [%]** |  |  | -0.52 | (-14.7%) | -0.55 | (-13.3%) | -0.50 | (+8.3%) | -0.49 | (+1.9%) |
| **relCt.Po_160µm_ [%]** |  |  | ns | | -0.46 | (-13.8%) |  |  |  |  |

Percent changes of the Pearson’s r are reported with respect to LEFT-LEFT regressions.

Coefficients are reported only for p-values < 0.05. * p < 0.01; ** p < 0.001. ns = loss significance
