# Supplementary material for: Large cortical bone pores in the tibia are associated with proximal femur strength
Source: PLoS One. 2019 Apr 17;14(4):e0215405. doi: 10.1371/journal.pone.0215405 (PMC6469812; doi:10.1371/journal.pone.0215405)
Supplement: S3 Table — Confidence intervals of the coefficients of correlation between tibial cortical bone vBMD and architecture with aBMDneck and proximal femur stiffness and strength. (DOC) [file pone.0215405.s005.doc]

|  | | | STANCE | | | | FALL | | | |
| --- | --- | --- | --- | --- | --- | --- | --- | --- | --- | --- |
|  | aBMD_neck_ | | hvFE_S | | hvFE_Fu | | hvFE_S | | hvFE_Fu | |
|  | 95% CIs of the Pearson r | | | | | | | | | |
| Left hip DXA (n=19) | | | | | | | | | | |
| aBMD_neck_ [mgHA/cm²] | / | | (0,22 | 0,84) | (0,43 | 0,89) | (0,30 | 0,86) | (0,50 | 0,91) |
| Left tibia (n=19) | | | | | | | | | | |
| vBMD_tot_ [mgHA/cm³] | (0,00 | 0,75) | (0,34 | 0,87) | (0,27 | 0,85) |  |  |  |  |
| vBMD_cort_ [mgHA/cm³] |  |  | (0,40 | 0,89) | (0,24 | 0,84) |  |  |  |  |
| SD(vBMD_cort_) [mgHA/cm³] |  |  | (-0,86 | -0,30) | (-0,82 | -0,18) |  |  |  |  |
| Tt.Ar [mm²] |  |  |  |  |  |  |  |  |  |  |
| Ct.Ar [mm²] | (0,07 | 0,78) | (0,19 | 0,83) | (0,38 | 0,88) | (0,17 | 0,82) | (0,20 | 0,83) |
| T.Ar [mm²] | (0,02 | 0,76) | (0,09 | 0,79) | (0,31 | 0,86) | (0,15 | 0,81) | (0,19 | 0,83) |
| Ct.Wba [%] | (0,08 | 0,79) | (0,46 | 0,90) | (0,41 | 0,89) |  |  | (0,03 | 0,77) |
| ROI_US_ | | | | | | | | | | |
| Ct.Th [mm] | (0,45 | 0,90) | (0,29 | 0,86) | (0,55 | 0,92) | (0,49 | 0,91) | (0,56 | 0,92) |
| Ct.Po [%] |  |  |  |  |  |  |  |  |  |  |
| Po.D [1/mm²] |  |  |  |  |  |  |  |  |  |  |
| Po.D_60µm_ [1/mm²] |  |  |  |  |  |  |  |  |  |  |
| Po.D_100µm_ [1/mm²] |  |  | (-0,80 | -0,11) | (-0,81 | -0,15) |  |  |  |  |
| Po.D_160µm_ [1/mm²] |  |  | (-0,79 | -0,08) | (-0,79 | -0,09) |  |  |  |  |
| relPo.n_60µm_ [%] |  |  |  |  |  |  |  |  |  |  |
| relPo.n_100µm_ [%] |  |  | (-0,79 | -0,09) | (-0,81 | -0,15) |  |  |  |  |
| relPo.n_160µm_ [%] |  |  | (-0,78 | -0,07) | (-0,79 | -0,09) |  |  |  |  |
| Po.Dm [mm] |  |  |  |  | (-0,76 | -0,01) |  |  |  |  |
| SD(Po.Dm) [mm] |  |  | (-0,80 | -0,12) | (-0,81 | -0,16) |  |  |  |  |
| Po.Dm_10%_ [mm] |  |  |  |  |  |  |  |  |  |  |
| Po.Dm_90%_ [mm] |  |  | (-0,77 | -0,05) | (-0,80 | -0,11) |  |  |  |  |
| Ct.Po_60µm_ [%] |  |  | (-0,76 | -0,01) | (-0,78 | -0,06) |  |  |  |  |
| Ct.Po_100µm_ [%] |  |  | (-0,78 | -0,06) | (-0,79 | -0,08) |  |  |  |  |
| Ct.Po_160µm_ [%] |  |  |  |  | (-0,76 | -0,02) |  |  |  |  |
| relCt.Po_60µm_ [%] |  |  | (-0,78 | -0,07) | (-0,83 | -0,20) | (-0,77 | -0,04) | (-0,78 | -0,06) |
| relCt.Po_100µm_ [%] |  |  | (-0,83 | -0,21) | (-0,84 | -0,25) | (-0,76 | -0,01) | (-0,77 | -0,03) |
| relCt.Po_160µm_ [%] |  |  | (-0,78 | -0,06) | (-0,79 | -0,10) |  |  |  |  |

Coefficients are reported only for p-values < 0.05. * p < 0.01; ** p < 0.001.
