# Supplementary material for: Large cortical bone pores in the tibia are associated with proximal femur strength
Source: PLoS One. 2019 Apr 17;14(4):e0215405. doi: 10.1371/journal.pone.0215405 (PMC6469812; doi:10.1371/journal.pone.0215405)
Supplement: S4 Table — Mean, Standard Deviation, ranges and coefficient of variation (CV) are reported for each variable together with the R2 and the p-value of the comparison (paired t-test or Wilcoxon signed rank test when parameters were not normally distributed) with the corresponding SAM measurement. For Cross-sectional areal properties for microCT are calculated dividing the corresponding volumetric measurement by the height (20 mm) of the analyzed stack. (DOC) [file pone.0215405.s006.doc]

|  |  |  | Comparison with SAM | |
| --- | --- | --- | --- | --- |
|  | Mean ± SD (min-max) | CV [%] | R² | p-val |
| Whole tibia properties (n=19) | | | | |
| Tt.Ar [mm²] | 441 ± 116 (336-859) | 26 | 0.99 | 0.629* |
| Ct.Ar [mm²] | 255 ± 64 (80-354) | 25 | 0.92 | 0.002 |
| T.Ar [mm²] | 270 ± 60 (125-378) | 22 | 0.97 | < 0.001 |
| Ct.Wba [%] | 57.0 ± 15.2 (16.7-75.9) | 27 | 0.93 | < 0.001 |
| ROI_US_ properties (n=19) | | | | |
| Ct.Th [mm] | 3.18 ± 1.03 (0.99-5.50) | 33 | 0.89 | 0.044 |

* Wilcoxon signed rank test
